# Epidemiological and Clinical Study of Infested Cases with *Pediculus capitis* and *P. corporis* in Khorasan-e-Razavi, Iran

**Published:** 2012

**Authors:** H Ramezani Awal Riabi, AR Atarodi

**Affiliations:** 1 Department of Health, School of Medicine, Gonabad University of Medical Sciences, Gonabad, Khorasan-e-Razavi, Iran; 2 Department of Basic Sciences, School of Paramedical Sciences, Gonabad University of Medical Sciences, Gonabad, Khorasan-e-Razavi, Iran

**Keywords:** *Pediculus capitis*, *Pediculus corporis*, Epidemiology, Treatment, Iran

## Abstract

**Background:**

Pediculosis (head lice) is considered as one of the most common health problems of the students in primary schools. The purpose of this study was to survey the prevalence rate of the infestation in the schools of Gonabad City (south of Khorasan-e-Razavi Province) to prevent its outbreak by on-time planning.

**Methods:**

In this retrospective-descriptive study, data were collected from the files of recorded health examinations of 55,997 female and male students of Gonabad City. We surveyed the infested cases to *Pediculus capitis* and *P. corporis* during 2006-2010. We used schools health unit of the city health center and review reports of infestation to head lice and body lice in cumulative centers.

**Results:**

The reports showed 398 cases of *P. capitis* and 3 *P. corporis* infestations, which 91.5% were female (*P* <0.05). Generally 46.4% were from rural and 63.6% were from urban areas (*P* <0.05). 71.3% of the infestation to head lice was from the last month and the rest had recently been infested. The most age group being infested were students of 6-10 years old and the lowest were >17 yr.

**Conclusion:**

Pediculosis infestation has become a major health problem in primary school students in south of Khorasan-e-Razavi.

## Introduction

Three types of lice found in human's body in arctic and tropical zones at the same time are head lice, body lice and pubic lice. The lice can infest anyone, the poor or the rich. These three types of lice have the property of blood-sucking and when feeding or moving in the skin, can cause skin irritation and itching at the site of blood-sucking (1, 2). Human lice are obligatory parasites which are wingless insects, with brown or gray color, existing from 9000 years ago. They have spread widely by people migration to different parts of the world. These two arthropods exclusively live on human and are not transferred by animals and plants (3).

Head lice, do not transmit any disease to human, only sucks humans' blood. This arthropod lays about 3-4 eggs on the hair around the ears and behind the neck every night, nits are hatched depending on temperature after one week and nymphs, get out and start blood-sucking within 24 hours to survive. The adult lice can live on the humans' hair for one month and if it is avoided from human body any way, it can not live more than 2 days (2).

Lice spread can be through direct contact of an uninfested person with an infested one. Some cases may be infested by using infested bed, couch, pillow, carpet, or using infested combs, brushes, towels or some wearing such as hats, scarves, coats, sport uniforms and hair ribbons worn by an infested person (4, 5). Instead of using expensive chemical pediculicides started from the 20^th^ century with the beginning of World War I, II and the pesticide DDT was used to control pediculosis infestation. Lindane insecticide was replaced for DDT in 1940 for its better performance and today pediculicide pyrethroids such as pyrethrin, permethrin, allethrin are used as drug therapy (3). Indiscriminate of pediculicide and non-normative usage of these insecticides have caused resistance among lice, and then has forced us to seek another compounds for removing the adult lice and their eggs (6).

Neem oil and tea-tree oil are from new plant compounds proposed for treatment of pediculosis, but little laboratory and clinical researches have been performed on these compounds (2). School children (3–5) are the most groups at risk of infestation that in this age, girls are more infested than boys because of their cover type and style, then combing the hair regularly and cleaning the hair is considered from the most appropriate control methods of infestation. The best time to prevent infestation is in opening time of the schools after summer vacation and when students return back to their schools and they should be examined for head lice infestation and the infested students should be treated by their parents (7).

This study was performed based on previous researches’ findings (8–11) and with the aim of identifying risk centers of epidemic pediculosis in schools and social areas intended to provide long-term treatment strategies for decreasing of lice-infestation burden in Gonabad City.

## Materials and Methods

### Study area

Gonabad City with an area of 10,000 square kilometers is located in south of Khorasan Razavi Province, 260 km far from Mashhad. It is located in east longitude of 46-57 to 27-59 and latitude of 30-34 to 34-54 with hot and dry climate and prone to tropical diseases prevalence including vector born diseases.

### Clinical examination

The study was conducted during 5 years (Mar 2006-Feb 2010) in primary, guidance and high schools as well as cumulative areas of Gonabad City. Female and male students were examined by trained health teachers under supervisory of entomologists considering medical laws and morality, students consent and confidentiality and respecting students' personality.

Demographic information of the infested students included: age, gender, geographical region and their history of infestation were recorded in the provided forms and their infestation type (Nit, nymph and adult) were determined, as well.

The students’ parents were emphasized to control and investigate their school children every day for 14 days and in the case of infestation treat them with permethrin1% shampoo. For treatment of the infested students, taking a shower was first proposed for washing their hairs with normal shampoo and combing their hairs after that, considering the length of the students' hairs, they were wet with permethrin1% shampoo and stained foamy for 8-10 minutes and then were washed and combed for treatment. They were advised to use the same shampoo in the case of observing infestation again after one week. It was emphasized to keep the infested members' own tools (such as hat, comb, neck scarf, scarf and so on) far or separated from the other members to prevent and save the other family members from infestation and wash them regularly, meanwhile the infested people were advised to get drug-therapy, too.

The second part of the study was calculating the prevalence rate of body lice infestation among students, residents of elderly nursing homes and students of dormitories. The underwear clothes of the target groups were checked by team members with complete satisfaction of the infested students and they were asked if they felt any itching on their body after obtaining written consent and informing them from the study aims.

### Statistical analysis

Data were analyzed by computer, using SPSS-12. Statistical analyses were performed by chi-square test with a significant *P* value of 0.05.

## Results

During a five-year study (2006-2010), 55997 female and male students were examined for pediculosis. Totally, 398 students were infested with head lice. The data were analyzed and compared in Table 1 based on gender, age, residential area and the infestation year.

**Table 1:** Epidemiological and clinical demography of *Pediculus capitis* in the city of Gonabad during 5 years (2006-2010)

Demographic Pediculosis	Total Pediculosis	Sex		Age Group (yr)								Geographic area		No. Background Risk	
Year		F	M	>17		11-17		6-10		6 <		Urban	Rural	No	Yes
				F	M	F	M	F	M	F	M				
2006	35	32	3	0	0	12	0	17	0	3	3	16	19	5	30
2007	70	62	8	2	0	19	1	24	1	17	6	56	14	15	55
2008	81	81	0	6	0	22	0	46	0	7	0	53	28	24	57
2009	113	109	4	6	4	39	0	47	1	17	2	39	33	27	86
2010	102	83	19	12	3	27	14	34	0	10	2	55	47	38	64
Total	401	367	34	26	7	119	15	168	2	54	13	219	141	109	292

Totally 91.5% were female and 8.5% were male students (*P* <0.05). 36.4% were in rural and 63.6% were in urban areas (*P* <0.05). 71.3% were infested to pediculosis from one month ago and the rest were recently infested. The most infested age group were young students (6-10 yr old), (98.7% girls and 1.3% boys) and the lowest age group were >17 yr (81.4% girls and 18.6% boys), respectively.

The prevalence of pediculosis infestation in different seasons was as follows: in spring 14%, in summer 3%, in autumn 59% and in winter 24%.

The most infestation to head lice was diagnosed in November of 2009. 96.5% of the cases were infested with nit and only 5% were infested with both nit and adult. No infestation was reported of fungus diseases resulted from head lice. The infestation with head lice was observed in one of the cumulative centers and in age group of over 50 years old with frequency of 3 cases, but it was not observed in students.

**Table 2:** The frequency of head lice infestation in schools of Gonabad City, based on month in the years 2006-2010

Mont Year	Mar	Apr	May	Jun	Jul	Aug	Sep	Oct	Nov	Dec	Jan	Feb
**2006**	0	0	0	0	0	0	0	18	2	15	0	0
**2007**	0	7	0	0	0	0	0	10	25	0	0	28
**2008**	5	0	0	0	4	0	0	21	25	7	10	9
**2009**	6	5	8	2	0	3	8	10	62	0	0	9
**2010**	0	7	8	0	0	1	3	24	28	18	7	6
**Total**	11	19	16	2	4	4	11	83	142	40	17	52

## Discussion

Pediculosis infestation has become a major health problem in our communities today with the development of urbanization, internal and external trips and transferred to schools and has encountered students with a social crisis. The primary school students of 6-10 years of age and female genders are the most groups involved in infestation with *P. capitis* (12).

The highest rate of infestation was also related to female genders (13–16) because of closely contact that primary school students may have with together. The infestation rate is higher in girls because of their covering type and their long hairs. The prevalence of *P. capitis* was 0.7% that has been compared with other studies such as: (1% in Shiraz, 3.6% in Tabriz and 3.8% in Kerman City) (17–19).

The best conditions that is suitable for lice reproduction is a temperature over 30 °C and dark environment away from light. One of the factors that affect the incidence of the infestation is the season of the year, since in warm seasons the growth of *P. capitis* will be prevented because of rising temperature over 36 °C, but in colder seasons wearing a hat or a scarf can provide a proper reproduction environment that in turn will cause the lice infestation in both male and female genders (2, 20).

According to the results of this study, pediculosis infestation in urban areas was higher than rural areas despite the fact that head lice should be higher in rural areas because of low levels of cultures and their life style and this may be due to the rising wave of jobseekers migration to the urban areas for employment. Another cause of this problem probably was migrating of village students to the city for continuing their education that this can also transfer infestation from village to the city (Table 1). In addition, busy working parents and not paying much attention to examinations of their school children, is not without any relation with pediculosis infestation as well; however no similarity was shown between our results and other studies (1).

We did not observe any difference between the effects of education level of students’ parents with pre-school students (21). Therefore human effort to reduce infestation's severity has led to production of chemicals against lice. Today after one or over a century, the use of insecticides, pediculicides, including permethrin1% in the form of drug shampoos is available in drug stores. But the indiscriminate and non-normative use of pediculicides has caused the immediate resistance of the arthropod (2, 22). And it is shown at the results that 70.3% have had previous infestation despite the use of this shampoo, their infestation was not treated and this could be because of the insufficient durability of permethrin on their hair (8-10 min) or because of little information of using drugs or loss of pediculicides property left against sun light and heat. Or it may be because of the lice own resistance to permethrin that should be studied.

It seems that training the parents of infested students can be effective on using drugs correctly and is a help to treat the other family members and to avoid the students from being infested again. In addition, the arbitrary use of these shampoos by, some of the individuals, due to the fear of stigmatization, will develop resistance in lice.

In *P. corporis* surveyed in this study, infested cases were newly entered to the other people environments and had stayed without examination and then as a result their severe infestation had affected the environment. For their treatment, the infested people were recommended to wear new and clean clothes and to destroy and burn all their infested clothes. The clothes, blankets, sheets, counterpane and pillow covers of all infested people were impregnated with permethrin shampoo and then they were washed after 4 to 5 hours and flattened in the sun.

After drying, they were heated with ironing to kill the rest nit. The living environment of the people were cleaned with a vacuum cleaner daily for one week with covering a mask and it was advised to examine new persons before entering the environment. The problem was surveyed after one month again and it was identified that *P. corporis* infestation had been removed by the performed method.

To treat an infested person with *P. capitis*, Vaseline oil, bitter almond oil, hair gel and eucalyptus were recommended for those who were forbidden to use these chemicals (such as pregnant mothers and infants), and it was found that they were effective and finally the infestation was removed that had also been proved in similar studies (23, 24). The impact of four shampoos such as d-phenothrin 0.2%, Lindane 1%, permethrin 1% and ordinary shampoo were tested on *P. capitis* and lindane 1% proved more efficient (25).

A Canadian study on the measurement of head lice resistance to permethrin showed that permethrin shampoo is effective on nit and adult lice (26), while benzyl benzoate does not eliminate the lice nit (13). In this study, hair length (to be long or short) and type (straight and curly) was effective in the amount of *P. capitis* infestation. Those with curly hair were less infested because straight hair save temperature more and provide a proper environment for lice growth (27, 28).

In conclusion, training of students’ parents is the best way to prevent epidemic of pediculosis in the community and in this way they will be more familiar with the lice infestation, preventing and the treatment in the family and community. This subject should be obvious for everyone that pediculosis infestation occurs in all communities and we should not label and brand each other badly since branding can cause hiding the infestation, then the most common cause of infestation spread in living environment is to hide it. Training others to use permethrin1% shampoo properly will be also effective in the prevention of lice resistance. It is necessary to measure and evaluate lice resistance to permethrin every 3 years.
